# The healthy ageing gene expression signature for Alzheimer’s disease diagnosis: a random sampling perspective

**DOI:** 10.1186/s13059-018-1481-6

**Published:** 2018-07-25

**Authors:** Laurent Jacob, Terence P. Speed

**Affiliations:** 1 Université de Lyon, Université Lyon 1, and CNRS, UMR 5558, Laboratoire de Biométrie et Biologie Evolutive, Villeurbanne, France; 2 Division of Bioinformatics, Walter and Eliza Hall Institute of Medical Research, and Department of Mathematics and Statistics, University of Melbourne, Melbourne, Australia

## Abstract

In a recent publication, Sood et al. (Genome Biol 16:185, 2015) presented a set of 150 probe sets that could be used in the diagnosis of Alzheimer’s disease (AD) based on gene expression. We reproduce some of their experiments and show that their signature is indeed able to discriminate between AD and control patients using blood gene expression in two cohorts. We also show that its performance does not stand out compared to randomly sampled sets of 150 probe sets from the same array.

**Electronic supplementary material:**

The online version of this article (10.1186/s13059-018-1481-6) contains supplementary material, which is available to authorized users.

Sood et al. built a signature by identifying 150 probe sets that predict chronological age on a gene expression dataset of muscle samples [1]. The 150 probe sets selected constitute the healthy ageing gene signature (HAGS) and were used in a 5-nearest-neighbor classifier to predict the chronological age or Alzheimer’s disease (AD) status of samples in other studies.

We focused on the AD status prediction experiments. We aimed to use the same labels and subset of samples from each cohort as used in Sood et al. [1] but cannot be certain as we do not have the authors’ code.

In their Figure 5, Sood et al. report areas under the receiver operating characteristic curve (AUCs) of 0.73 and 0.66 using the HAGS for AD in cohorts 1 and 2, respectively [1]. We estimate the AUC of two 5-nearest-neighbor classifiers by leave-one-out cross validation (LOOCV) on a randomly sampled 50% of each dataset (stratified by status). One classifier uses the HAGS and the other one uses a randomly sampled 150 probe sets. We repeat the operation 1000 times, using a new random selection of probe sets for each repetition. More details of our experiments including patient selection, grouping, and sampling schemes are available in Additional file 1. We also provide the R code used in these experiments as Additional file 2.

Figure 1 shows that the distribution of the performance obtained by the HAGS and by random sets of 150 probe sets are very similar. This suggests that we should expect similar AD status prediction performance for the HAGS and random sets of probes on average for patients from the same distributions of the phenotype, conditional to the expression of all probes, as these cohorts.

**Fig. 1:**
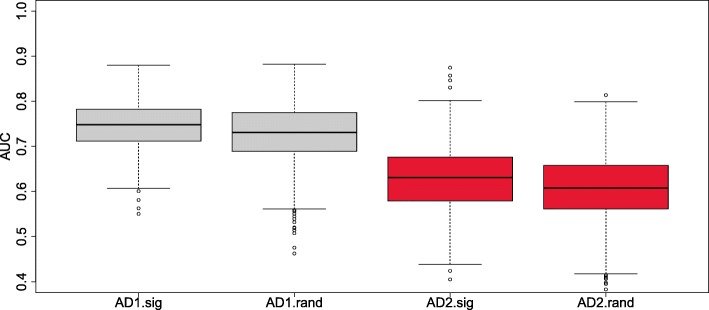
Area under the receiver operating characteristic curves. This was obtained by LOOCV of a 5-nearest-neighbor classifier over 1000 random selections of 50% of the arrays, using the HAGS probe sets (.sig suffix) and a new random selection of 150 probe sets each time (.rand suffix), over the two AD cohorts. AD Alzheimer’s disease, AUC area under the receiver operating characteristic curve, HAGS healthy ageing gene signature, LOOCV leave-one-out cross validation

We also assessed whether the HAGS stands out from random signatures by looking at its median performance across random samplings from the cohorts. We drew 500 random sets of 150 probe sets, and used each of these random sets on the same 200 stratified samplings of 50% of the cohorts. If each of the 500 sets of 150 probe sets performs well by chance on a few of the 200 sub-samplings but performs poorly on the others, we would expect the median AUC of the HAGS across the 200 subsamples to stand out from the distribution of median AUCs obtained using the 500 random sets of probe sets. Figure 2 shows that this is not the case: the median AUC obtained using the HAGS lies within the interquartile range of the median AUCs obtained using random sets of probe sets.

**Fig. 2:**
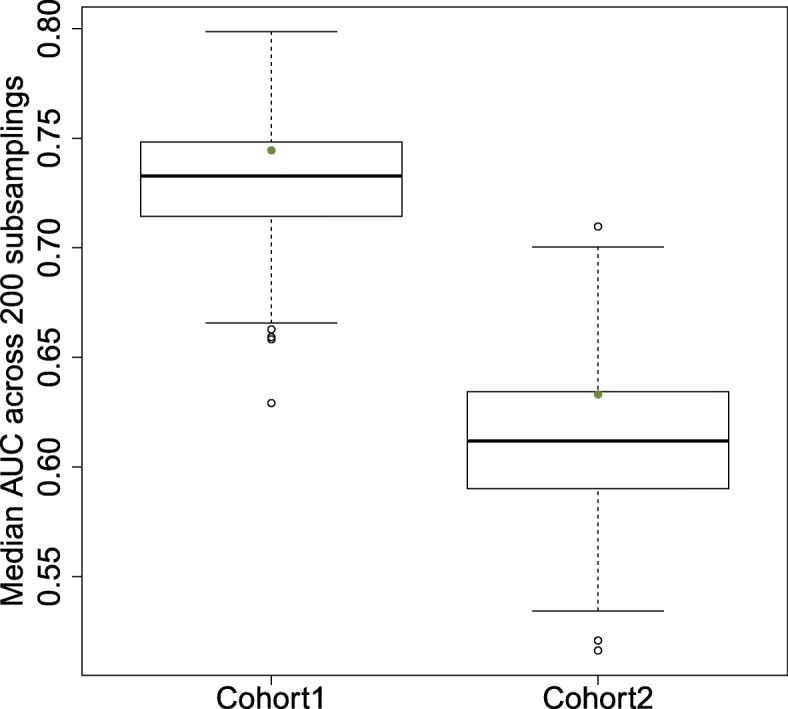
Median area under the receiver operating characteristic curves. This was obtained by LOOCV of a 5-nearest-neighbor classifier across 200 random selections of 50% of the arrays, using the HAGS probe sets (green dots) and 500 random selections of 150 probe sets (box plots), over the two AD cohorts. AD Alzheimer’s disease, AUC area under the receiver operating characteristic curve, HAGS healthy ageing gene signature, LOOCV leave-one-out cross validation

That the random probe sets perform as well as a set of probes that were selected for their predictive power on a different dataset is not too surprising. Ein-Dor et al. noted that sampling from a small set of arrays leads to the selection of different gene expression signatures for breast cancer prognosis [2]. Haury et al. found no significant difference between the AUCs obtained using random signatures and signatures selected for their predictive performance [3]. Our finding that randomly selected sets of probes perform as well as the HAGS on average is consistent with their observation.

The AUCs published in Sood et al. [1] are the product of two factors: the predictive value of the 150 probe sets selected (HAGS) and the difficulty of the prediction problems on which they are assessed: discriminating between 25- and 65-year-old patients or between control and AD patients on these particular datasets. Our random sampling experiments suggests that the AUCs presented are not exceptionally high given the intrinsic difficulty of the prediction problems. In particular, there is no reason to believe that the selection protocol (identifying genes that discriminate 15 healthy young from 15 healthy old patients) picked up an exceptionally predictive signal for healthy ageing.

A principal component analysis of either cohort actually reveals that the first principal component explains about 25% of the total variance and separates the two status groups rather well. A possible explanation is an unobserved confounding variable associated with both gene expression measurements and AD status. Another possibility is that the problem of discriminating between controls and patients diagnosed with AD from blood gene expression is actually a feasible one because the presence of AD at this stage has a sufficiently strong effect on the overall gene expression. In this case, the question moves to deciding whether a good predictor of current AD status is also a good predictor of future AD status. The latter is arguably a more important objective [4], allowing mass population screenings to detect those at risk, but could prove more difficult than the former as it may be associated with more subtle effects on gene expression.

Our discussion underscores the importance of considering random sampling perspectives when building a gene signature, especially when interpreting its content or studying its overlap with other signatures, not just its predictive power.

## Additional files

Additional file 1 Supplementary material. Detailed explanations of the experiments presented in this correspondence. (PDF 81 kb)

Additional file 2 Code. This R code can be used to generate all figures presented in this correspondence. (TGZ 81 kb)
