# Supplementary material for: The healthy ageing gene expression signature for Alzheimer’s disease diagnosis: a random sampling perspective
Source: Genome Biol. 2018 Jul 25;19:97. doi: 10.1186/s13059-018-1481-6 (PMC6060554; doi:10.1186/s13059-018-1481-6)
Supplement: Supplementary file 1 — Supplementary material. Detailed explanations of the experiments presented in this correspondence. (PDF 81 kb) [file 13059_2018_1481_MOESM1_ESM.pdf]

Supplementary material to: "The healthy ageing gene expression signature for Alzheimer's disease diagnosis: a random sampling perspective"

Laurent Jacob Terence P. Speed

July 5, 2017

1 Summary of the experiments in [1]

The first part of the experiments in [1] builds their signature and assesses its ability to predict chronological age in different settings. This first part involves eight gene expression studies: six from muscle, one from brain and one from skin samples. The skin study was done on Illumina Human HT-12 V3 arrays and all the others on Affymetrix HGU133plus2 arrays. The first muscle dataset involves muscle samples from 15 young and 15 old healthy individuals and is only used to build the signature. The selection process retains probe-sets which are both differentially expressed between young and old samples as measured by limma [2] and predictive of chronological age in the context of a 5 nearest neighbor classifier, along with other selected probe-sets. The 150 probe-sets selected constitute the healthy ageing gene signature (HAGS) and they are then used in a 5 nearest neighbor classifier to predict the chronological age of samples in the other studies; the study used to select the signature is not used any more in the rest of their experiments. [1] use two different protocols to evaluate the prediction performance. For all except the skin data, they use external validation: the samples from one of the muscle studies (Campbell) are used as neighbors to predict the age of the tested samples in the four remaining muscle and the brain study. For the skin study, they use leave one out cross validation (LOOCV). They also use LOOCV on two of the muscle studies and the brain study to produce ROC curves on their Figure 2. They obtain reasonably high AUCs and conclude that their 150 probe-sets are predictive of chronological age regardless of the tissue and platform.

In the last part of their experiments, the 150 HAGS probe-sets are used to predict Alzheimer's Disease (AD) status from blood gene expression samples in two cohorts. Gene expression is measured using Illumina Human HT-12 V3 and V4 arrays respectively. Samples are selected within each cohort to make AD status independent of potential confounders such as age, gender or ethnicity. The 150 probe-sets are mapped

to Illumina probe-sets. They lead to LOOCV Areas Under the ROC Curve (AUCs) of 0.73 and 0.66.

In our correspondence, we attempt to reproduce some of the experiments from [1] using R 3.3. We use the 5 nearest neighbor algorithm as implemented in the `class` package. AUCs are measured using the `ROCR` package. We download the Affymetrix arrays (GSE59880) and Illumina expression beadchips (GSE63060, GSE63061) available on GEO, using `GEOquery`. We normalize the Affymetrix arrays using RMA as implemented in `affy`. We cannot be certain that our protocol is identical to the one of [1] as we do not have access to their code, but we followed the description provided in the article and obtained similar AUCs.

2 Experiments on the AD cohorts

This section provides details on the protocol used for the experiments presented in the correspondence.

2.1 Patient selection

We aim at using the same subset of samples from each cohorts as used in [1] but cannot be certain as it is unclear which samples in the AD cohorts were used in the original paper.

More precisely, we retain the samples from the two cohorts deposited in GEO (GSE63060 and GSE63061) which are annotated as ”included in case -control study: yes”. This yields 155 and 142 samples, consistent with the total 297 reported in the Materials and Methods section of [1]. The samples stratify as 49 ”AD”, 67 ”CTL” and 39 ”MCI” (resp. 40 ”AD”, 72 ”CTL”, 30 ”MCI”), which also matches the numbers provided in Material and Methods by [1].

We note that the authors used a different selection of 113 and 111 patients in the Results section for their ranking experiment, which probably correspond to ”AD” and ”control AD” in Table 1. However the only existing statuses in the first cohort are ”AD”, ”CTL” and ”MCI”. We did not find any specific information on patient selection for ROC analysis, and assume the authors used the 155+142 selection reported in Materials and Methods.

2.2 Patient grouping

We do not have access to the precise labels used for the ROC analysis of [1]. Using their signature and 5-nn to predict CTL vs AD+MCI, we obtain similar LOOCV AUCs (0.76 and 0.66) to those they report (0.73 and 0.66). We also notice that AD and MCI have very similar projections along PC1 (Figure 1, where PCs where computed using the `svd` function of R after mean-centering the data). We therefore use this setting in our

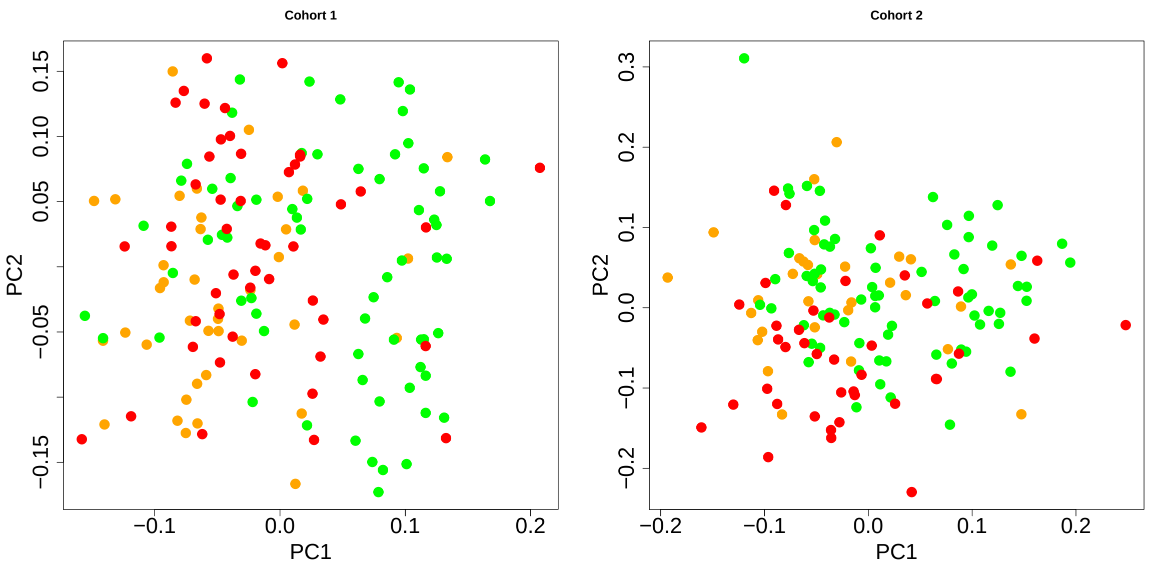

Figure 1: Projection of blood expression samples onto their first two principal components for cohort 1 (left) and 2 (right). Red dots are AD patients, orange ones are MCI and green ones are controls.

experiments and create two classes by merging the MCI (mild cognitive impairment) and AD status – we refer to this merged class as AD in the remainder of this discussion.

2.3 Sampling

We sample 150 probe-sets uniformly from the 54675 present on the Affymetrix Human Genome U133 Plus 2.0 Array used in the dataset available on GEO (GSE59880) and used in [1] to build their 150 probe-set signature. We then map these 150 probe-sets to the Illumina HumanHT-12 V3.0 and V4.0 expression beadchips used for cohort 1 and 2 respectively by extracting gene symbols for the sampled probe-sets using the `hgu133plus2.db` R package, and using all Illumina probes associated with the same gene symbol as indicated by the `illuminaHumanv3.db` and `illuminaHumanv4.db` R packages.

We stratify our array sampling by status to make sure that the proportion of AD and control status remains unchanged. More precisely, we sample 50% of the arrays withing the AD class and 50% of the arrays within the control class. Further stratifying by age and gender leads to similar result.

References

[1] Sanjana Sood, Iain J Gallagher, Katie Lunnon, Eric Rullman, Aoife Keohane, Hannah Crossland, Bethan E Phillips, Tommy Cederholm, Thomas Jensen, Luc J C

van Loon, Lars Lannfelt, William E Kraus, Philip J Atherton, Robert Howard, Thomas Gustafsson, Angela Hodges, and James A Timmons. A novel multi-tissue rna diagnostic of healthy ageing relates to cognitive health status. *Genome Biol*, 16:185, 2015.

[2] Matthew E Ritchie, Belinda Phipson, Di Wu, Yifang Hu, Charity W Law, Wei Shi, and Gordon K Smyth. limma powers differential expression analyses for rna-sequencing and microarray studies. *Nucleic Acids Res*, 43(7):e47, Apr 2015.
