# Supplementary figures and images for: The healthy ageing gene expression signature for Alzheimer’s disease diagnosis: a random sampling perspective
Source: Genome Biol. 2018 Jul 25;19:97. doi: 10.1186/s13059-018-1481-6 (PMC6060554; doi:10.1186/s13059-018-1481-6)

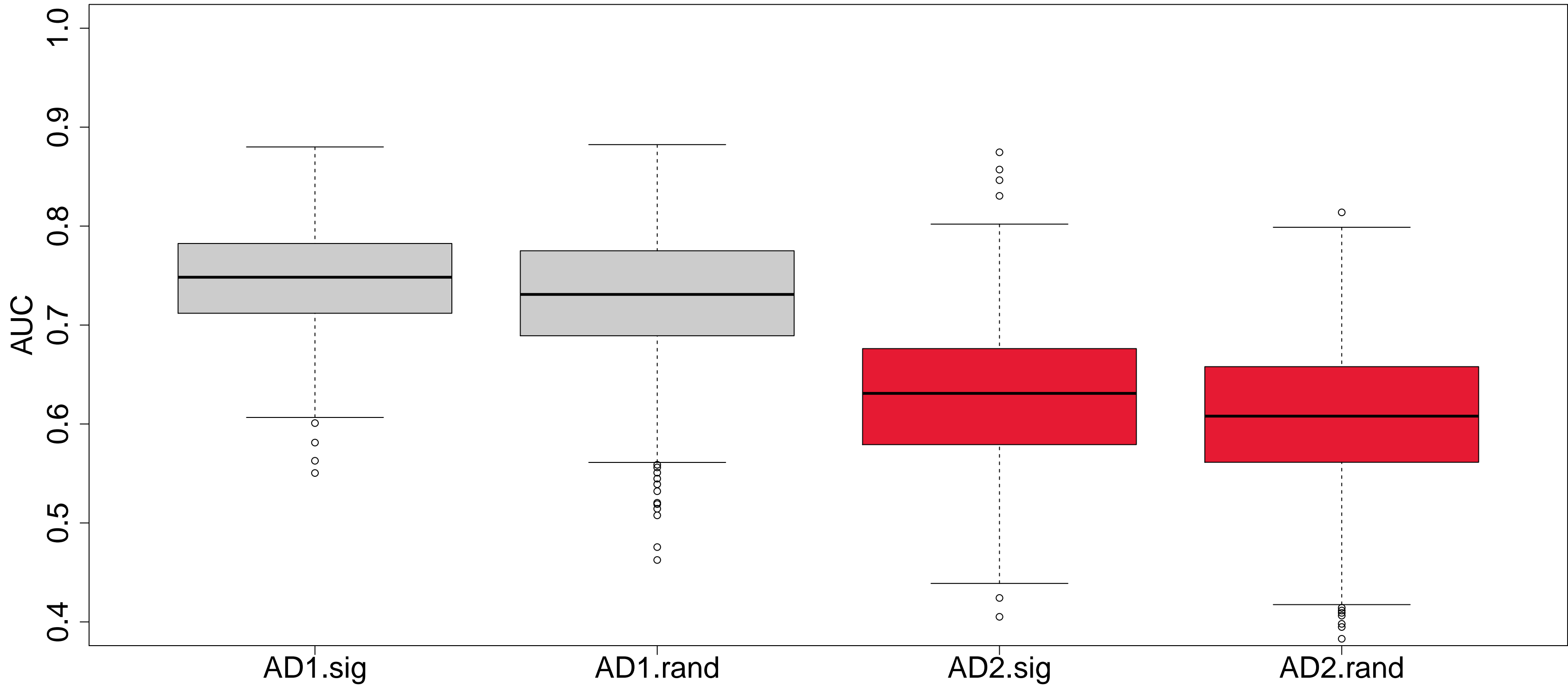

Supplement: Supplementary file 2 — Code. This R code can be used to generate all figures presented in this correspondence. (TGZ 81 kb) [file 13059_2018_1481_MOESM2_ESM.tgz › BMC-randomSampling/plots/BMC-Fig1-AD-subsample.pdf]

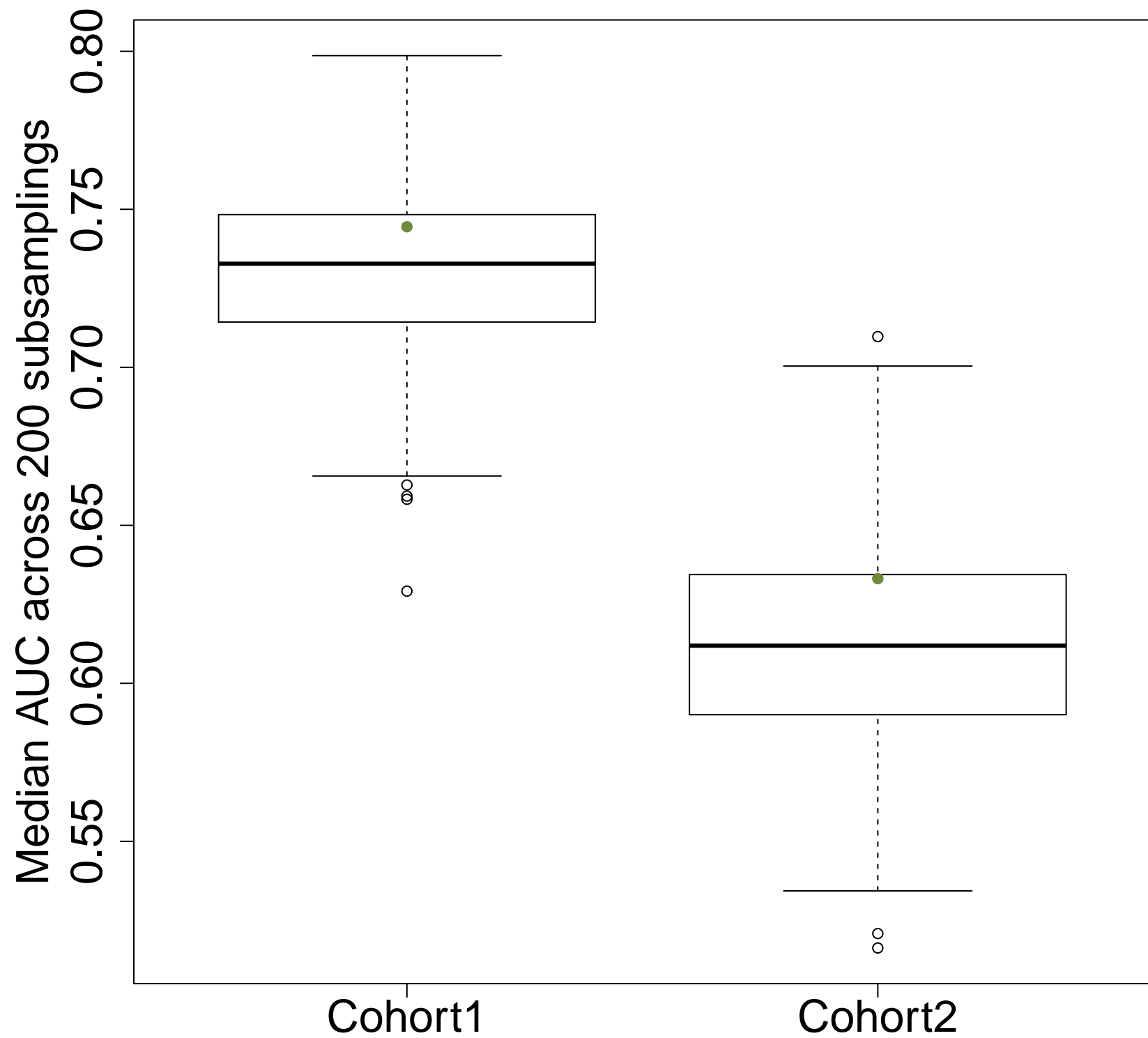

Supplement: Supplementary file 2 — Code. This R code can be used to generate all figures presented in this correspondence. (TGZ 81 kb) [file 13059_2018_1481_MOESM2_ESM.tgz › BMC-randomSampling/plots/BMC-Fig2-AD-subsample-bySig.pdf]
